# Experiencing statistical information improves children’s and adults’ inferences

**DOI:** 10.3758/s13423-022-02075-3

**Published:** 2022-06-01

**Authors:** Christin Schulze, Ralph Hertwig

**Affiliations:** Center for Adaptive Rationality, Max Planck Institute for Human Development, Lentzeallee 94, 14195 Berlin, Germany

**Keywords:** Description–experience gap, Conjunction rule, Bayesian probability updating, Statistical intuitions

## Abstract

How good are people’s statistical intuitions? Recent research has highlighted that sequential experience of statistical information improves adults’ statistical intuitions relative to situations where this information is described. Yet little is known about whether this is also the case for children’s statistical intuitions. In a study with 100 children (8–11 years old) and 100 adults (19–35 years old), we found that sequentially experiencing statistical information improved both adults’ and children’s inferences in two paradigmatic reasoning problems: conjunction and Bayesian reasoning problems. Moreover, adults’ statistical competencies when they learned statistical information through description were surpassed by children’s inferences when they learned through experience. We conclude that experience of statistical information plays a key role in shaping children’s reasoning under uncertainty—a conclusion that has important implications for education policy.

Accurate statistical intuitions are crucial for coping with the demands of an uncertain world. How do people make statistical inferences, and how good are their statistical intuitions? Recent research has highlighted that feedback, learning opportunities, and firsthand experience with statistical information elicit different judgments and decisions than do symbolic descriptions of this information. In risky choice, for instance, people make systematically different choices in description-based monetary gambles than they do when they learn about outcomes and their relative frequencies from experience (for reviews of this *description–experience gap*, see Hertwig & Erev, 2009; Rakow & Newell, 2010; for a meta-analysis, see Wulff et al., 2018). Description–experience gaps have also been demonstrated in categorization (Nelson et al., 2010), social interaction in strategic games (Martin et al., 2014), decisions under ambiguity (Dutt et al., 2014; Güney & Newell, 2015), causal reasoning (Rehder & Waldmann, 2017), intertemporal choice (Dai et al., 2019), and, importantly, statistical intuitions (Lejarraga & Hertwig, 2021; Schulze & Hertwig, 2021).

Increasing the level of experiential involvement is thought to improve people’s statistical intuitions (e.g., Schulze & Hertwig, 2021). In this context, it is helpful to understand description and experience not as dichotomous but as spread across a continuum (e.g., Rakow & Newell, 2010; Schulze & Hertwig, 2021). Accordingly, described frequency formats that reflect part of the underlying original experience (i.e., a series of events) have been shown to improve several types of inferences, including Bayesian inferences (Gigerenzer & Hoffrage, 1995; McDowell & Jacobs, 2017) and inferences about conjunctive events (Fiedler, 1988; Hertwig & Gigerenzer, 1999; Tversky & Kahneman, 1983). Moreover, Hogarth and Soyer (2011) showed that offering individuals the opportunity to directly experience simulated outcomes of probabilistic processes in otherwise description-based inference tasks (e.g., conjunction problems, Bayesian problems, the Monty Hall problem) improved the inferences of both naïve and statistically versed individuals. Furthermore, Armstrong and Spaniol (2017) found that Bayesian inferences made on the basis of experience resulted in more accurate estimates of posterior probabilities than when a described format was used (see also Wegier & Shaffer, 2017).

Although previous work has garnered much insight into how experience shapes adults’ statistical intuitions, little is known about the link between children’s statistical intuitions and the sequential experience of statistical information. Previous research has mainly focused on children’s reasoning in either description-based or experience-based situations. In this article, we directly compare children’s and adults’ statistical reasoning in paradigmatic inference problems—conjunction problems and Bayesian problems—in description-based and experience-based formats.

## Children’s statistical inferences in description and experience

Conjunction and Bayesian problems are paradigmatic reasoning problems that have been studied with children and that, in described formats, even adults tend to fail (e.g., Gigerenzer & Hoffrage, 1995; Tversky & Kahneman, 1983). In classic conjunction problems, a stereotypical description of a hypothetical person and their interests or characteristics is provided, and participants judge the likelihood of attributes that either fit or do not fit the described stereotype as well as the conjunction of attributes (e.g., Davidson, 1995; Tversky & Kahneman, 1983). Participants tend to judge the conjunction of stereotypical and nonstereotypical attributes as more probable than nonstereotypical attributes, thus violating the conjunction rule—a judgment often referred to as the conjunction fallacy. When 9-year-olds were familiar with the stereotype invoked by a problem, comparable rates of conjunction-rule violations were found in both children and adults (Morsanyi et al., 2017). Although the role of experience in children’s reasoning about conjunctive probabilities has not yet been investigated, a closely related statistical competency—the ability to solve class inclusion problems—suggests that experience plays an important role. Class inclusion problems, like conjunction problems, require that inclusion relationships be considered (Reyna, 1991) and are readily solved by children of a certain age (see, e.g., Winer, 1980). Importantly, the two types of problems differ in representation format: Class inclusion problems typically present information about frequencies in analogical pictorial formats, whereas description-based conjunction problems rely on probability formats that do not “leak” numerosity information.

Bayesian reasoning requires individuals to update their beliefs about uncertain events in light of new data—a key competence within the rational choice framework. In a typical description-based problem, participants provide an exact Bayesian posterior probability based on relevant statistics presented as conditional probabilities (e.g., Gigerenzer & Hoffrage, 1995). Solving such problems is a challenging task; no children aged 9–12 years (Zhu & Gigerenzer, 2006), and an estimated 4% of adults (McDowell & Jacobs, 2017) correctly solved this type of problem. However, when the statistical information was presented in a natural frequency format—thus retaining more of the underlying experience—adults’ Bayesian inferences improved considerably (Gigerenzer & Hoffrage, 1995; McDowell & Jacobs, 2017), and 12-year-olds’ inferences were comparable to those of adults in a conditional probability format (Zhu & Gigerenzer, 2006; but see Pighin et al., 2017). Using nonnumeric icon array representations of natural frequencies, Gigerenzer et al. (2021) showed that children as young as 7 years old have accurate Bayesian intuitions: Second-graders solved 22% to 32% of all Bayesian problems; fourth-graders solved 50% to 60%. Finally, Girotto and Gonzalez (2008) tested children’s ability to qualitatively update predictions based on new evidence, without needing to provide an exact posterior probability. They found that from the age of 5, children accurately revised their predictions consistent with the normative Bayesian standard when they were given new information about a drawn sample verbally.

Although this previous developmental work on Bayesian reasoning involves features that reflect some of the underlying experience, natural frequencies and icon arrays are nevertheless descriptive formats. Experience goes beyond the presentation of summed frequencies, static pictorial representations, or verbal descriptions of samples: It typically involves interaction with the environment to understand its statistical structure—for instance, by sampling information sequentially (Hertwig et al., 2018; Schulze & Hertwig, 2021). Several studies have investigated the benefits of experiential sampling for adults’ ability to solve statistical inference problems (e.g., Armstrong & Spaniol, 2017; Hogarth & Soyer, 2011; Wegier & Shaffer, 2017) and to make choices that satisfy Bayes’s theorem (Domurat et al., 2015), but no developmental work directly investigates the effect of sequential sampling.

To fill this gap, we evaluated the role of sampling experience in improving children’s statistical reasoning, by pitting children’s and adults’ inferences in experience-based conjunction and Bayesian problems against their description-based reasoning. To compare text-based descriptions of statistical information to the sequential experience thereof, we focused on children aged 8 to 11 years, as they were able to read description-based problems. This age range is comparable to those in previous developmental studies investigating children’s ability to reason in accordance with Bayes’s theorem and the conjunction rule (e.g., Davidson, 1995; Gigerenzer et al., 2021; Morsanyi et al., 2017; Zhu & Gigerenzer, 2006). Moreover, to connect with seminal work on the role of experience in improving adults’ statistical intuitions, we followed the approach taken by Hogarth and Soyer (2011), in which sampling experience is added to a summary description. This setup allowed us to explore whether experience can help children overcome the nontransparency of equivalent descriptions.

How will sampling experience affect children’s statistical reasoning compared to description? Recent research on the development of statistical intuitions in infancy suggests a beneficial effect of experience for children’s inferences and has shown that babies possess a remarkable ability to draw accurate statistical inferences from finite samples (for a review, see Denison & Xu, 2019). For example, infants infer the properties of populations from a randomly drawn sample (Xu & Garcia, 2008), integrate physical information about objects when making statistical inferences (Téglás et al., 2007), and take into account attributes of sampling agents (Xu & Denison, 2009). Comparable statistical inference abilities have been found in humans’ closest relatives, great apes, and even in birds (e.g., Bastos & Taylor, 2020; Eckert et al., 2018; Rakoczy et al., 2014). Because infants and nonhuman animals can neither produce nor process symbolic descriptions of the world, their statistical intuitions have been studied in paradigms that involve experiencing statistical information. In a recent review, we showed that the distinction between described and experienced statistical information is crucial to understanding seemingly puzzling differences in infants’ and adults’ abilities to reason statistically (Schulze & Hertwig, 2021). The experimental paradigms used with infants, animals, and adults also differ in other ways (for a summary of further, complementary factors that may play a role, see Schulze & Hertwig, 2021), but the description–experience distinction is a thread running through research on statistical intuitions and its surprisingly incongruent results and conclusions (see also Lejarraga & Hertwig, 2021). We now turn to an experiment that aims to bridge research on the development of statistical intuitions in early childhood with research on the role of experience in improving adults’ statistical intuitions.

## Experiment: Description and experience across development

We presented children and adults with two types of reasoning problems—conjunction problems and Bayesian problems—in either an experience-based or a description-based format. Various experimental paradigms have been used to study children’s ability to make accurate Bayesian inferences (e.g., Gigerenzer et al., 2021; Girotto & Gonzalez, 2008; Pighin et al., 2017; Zhu & Gigerenzer, 2006) and their inclination to violate the conjunction rule (e.g., Davidson, 1995; Morsanyi et al., 2017; Morsanyi & Handley, 2008). With the exception of Zhu and Gigerenzer (2006), who compared children’s Bayesian inferences in a natural frequency and a conditional probability format, the experimental procedures of these prior studies did not vary the degree of experiential involvement. To identify conjunction and Bayesian problems best suited for both children and adults, we conducted a pilot study in which we tested conjunction and Bayesian problems in a frequency format paired with icon arrays; Appendix 1 summarizes method and results.

## Method

### Participants

We recruited 100 children (ages 8–11 years, mean age 9.43 years, 50 female) and 100 adults (ages 19–35 years, mean age 26.16 years, 57 female) via the subject pool of the Max Planck Institute for Human Development and at the Natural History Museum in Berlin, Germany. A power analysis, based on the effect sizes reported in studies examining related forms of experiential involvement (e.g., natural frequency formats; McDowell & Jacobs, 2017), informed sample size prior to recruitment (using G*Power software; Faul et al., 2007). Fifty participants in each age group were assigned to each of two between-subjects conditions: a description-based probabilities format and an experience-based sampling format.^1^ Participants received a performance-based payment (earning €1 for each correct inference), and an additional flat fee (children and parents who traveled to the Max Planck Institute for Human Development received €10; all others received €5). The experiment was reviewed and approved by the institutional review board of the Max Planck Institute for Human Development and participants gave informed consent prior to taking part in the study.

### Materials

The experiment was administered as a series of paper-and-pencil questions. Participants made a total of four inferences on two conjunction problems and two Bayesian problems (the order of which was counterbalanced across participants). The conjunction problems were based on child-friendly versions of the Linda problem (adapted from Morsanyi et al., 2017; Morsanyi & Handley, 2008); the Bayesian problems were child-friendly versions adapted from Zhu and Gigerenzer (2006). Appendix 2 lists all inference problems used in their description-based and experience-based formats.

**Fig. 1:**
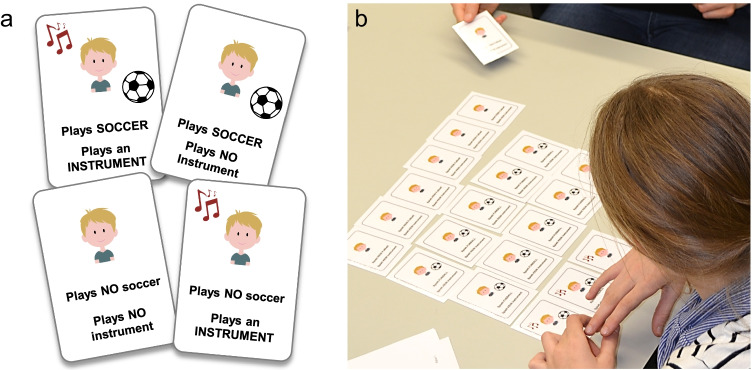
Illustration of **a** sampling cards and **b** sampling procedure used in the experience-based conditions of the experiment. The parent of the depicted participant gave written consent for the photo to be published in this article

In the description-based problems, participants ranked hypotheses according to their probability of being true in the conjunction problems (see Tversky & Kahneman, 1983) and provided posterior probability estimates in the Bayesian problems (e.g., Gigerenzer & Hoffrage, 1995; see Appendix 2). In the experiential format, participants experienced sequential sampling of all elements featured in the problem (see, e.g., Armstrong & Spaniol, 2017), then provided a response in a frequency format (see Appendix 2; e.g., Gigerenzer & Hoffrage, 1995; Hertwig & Gigerenzer, 1999). The sampling procedure is illustrated in Fig. 1 and was carried out with four sets of 9 × 6-cm custom-made playing cards, one for each problem (see Vallée-Tourangeau et al., 2015). The number of cards in each problem matched the frequencies stated in the scenario. We implemented this card-based sampling procedure (rather than a computerized form of simulated experience; e.g., Hogarth & Soyer, 2011) to make the task appealing for children, reduce memory load (the entire sample remained available), and allow participants to directly engage with the information (e.g., handling and sorting the cards).

### Procedure

Participants were tested individually. Adults in the description-based condition completed the task by themselves; for children and in the experiential adult condition, the experimenter was present during the experiment. All participants were informed that they would be presented with a series of puzzles and that they could earn additional money for correct responses. In the conditions in which the experimenter was present, participants sat at a table with the experimenter at an adjacent side, facing the participant (see Fig. 1b). The experimenter read each problem aloud, encouraging participants to read along, ask questions if they had any, and take as much time as needed to solve each problem to the best of their ability.

The sampling procedure started with the experimenter placing a deck of cards on the table and revealing four cards that showed all possible outcomes in the scenario (see Fig. 1a). The experimenter then returned the four cards to the deck, shuffled all the cards, and drew cards one by one, placing each on the table in front of the participant and verbalizing the information shown (or engaging the participant to do so). The cards were positioned in groups of each of the four possible outcomes in a predetermined order to facilitate understanding. In the Bayesian problems, the cards were placed in the order of the information listed in the scenario (see Appendix 2). In the conjunction problems, cards showing the conjunctive event were placed between cards showing the stereotypical and nonstereotypical constituents (see Fig. 1b).

### Data analysis

All data are available via the Open Science Framework and can be accessed at https://osf.io/ajb3v/. In addition to conventional methods of null-hypothesis significance testing, we conducted Bayesian statistical analyses, based on Bayesian contingency analyses using independent multinomial sampling (Jamil et al., 2017). For these analyses, we report Bayes factors, denoted as *BF*_10_, that quantify the strength of evidence in favor of the alternative hypothesis, where *BF*_10_ > 1 indicates support for the alternative hypothesis and *BF*_10_ < 1 indicates support for the null hypothesis. Conventionally, a *BF*_10_ between 3 and 20 (0.33–0.05) is interpreted as indicating positive evidence for the alternative (null) hypothesis, 20 to 150 (0.05–0.0067) as strong evidence, and greater than 150 (<0.0067) as very strong evidence (Kass & Raftery, 1995). All Bayes factors were estimated in JASP (Version 0.16; JASP Team, 2021).

## Results

**Fig. 2:**
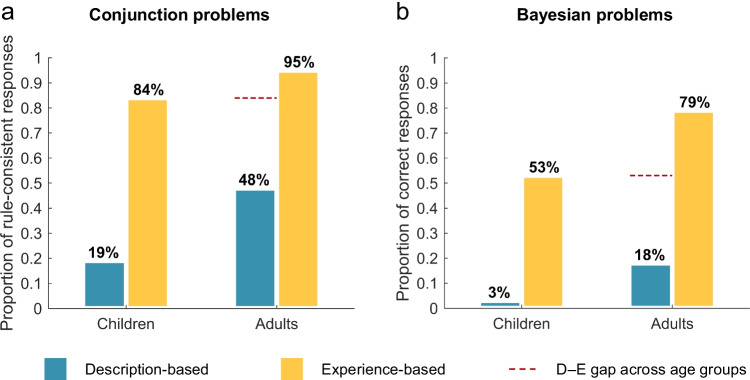
Proportion of **a** responses consistent with the conjunction rule and **b** correct Bayesian responses for children and adults in experience-based and description-based reasoning conditions. Dashed lines compare children’s statistical inferences in experience with adults’ inferences in description and show a description–experience (D–E) gap across age groups (see Discussion)

Figure 2a shows the proportion of responses consistent with the conjunction rule (i.e., the probability/frequency of both constituents is higher than, or equal to, that of the conjunctive event; see Fiedler, 1988) given by children and adults in description-based and experience-based representation formats, respectively. The large majority of adults (97%) and children (87%) judged the stereotypical constituents to be most probable/frequent on both conjunction problems, indicating a general familiarity with the invoked stereotypes.^2^ Figure 2b shows the proportion of correct Bayesian responses given by children and adults in each representation format. We used a strict outcome criterion to evaluate responses and counted only those as correct that numerically exactly matched the solution prescribed by Bayes’s theorem (rounded to the nearest integer; see Zhu & Gigerenzer, 2006).

When the information was conveyed in a description-based probability format, overall, adults correctly solved 18% of the Bayesian problems and gave rule-consistent responses for 48% of the conjunction problems. Children solved fewer problems in accordance with the statistical rule: 3% of the Bayesian problems and 19% of the conjunction problems.^3^ When they sequentially experienced the statistical information, both adults’ and children’s inferences improved considerably: Overall, adults solved 79% of Bayesian problems and 95% of conjunction problems; children solved 53% and 84%, respectively.

To quantify the associations between participants’ statistical intuitions and the level of experiential involvement, we analyzed participants’ number of correct Bayesian responses and responses consistent with the conjunction rule for each age group and type of reasoning problem separately, as shown in Table 1. Striking description–experience gaps emerged for both age groups and both types of reasoning problems. In fact, across age groups, the odds of not violating the conjunction rule on any problem were 12.59 times higher in experience than in description; the odds of correctly solving all Bayesian problems were 32.33 times higher in experience than in description.

**Table 1:** Number of children’s and adults’ correct Bayesian responses and responses consistent with the conjunction rule, and statistical evaluation of the associated description–experience gaps

Age group Reasoning problem	Experiential involvement	Number of correct/rule-consistent responses			Description–experience gap		
		0/2	1/2	2/2	*χ*^2^	*p*	*BF*_10_
Children (8–11 years)							
Conjunction problem	Description	34	13	3	60.28	<.001	5.61×10^14^
	Experience	0	16	34			
Bayesian problem	Description	47	3	0	46.49	<.001	8.53×10^9^
	Experience	14	19	17			
Adults (19–35 years)							
Conjunction problem	Description	22	8	20	32.31	<.001	8.10×10^6^
	Experience	0	5	45			
Bayesian problem	Description	35	12	3	49.68	<.001	9.68×10^10^
	Experience	4	13	33			

*Note.* For all chi-square tests *df* = 2. *BF*_10_ denotes Bayes factors that quantify the strength of evidence in favor of the alternative hypothesis (*BF*_10_ > 1 indicates support for the alternative hypothesis; *BF*_10_ < 1 indicates support for the null hypothesis). Bayes factors were estimated under the assumption that rows were sampled as independent multinomials with their total fixed (see Jamil et al., 2017)

## Discussion

Even the youngest learners regularly face uncertain consequences and statistical information. How do children learn to deal with an uncertain world and what role does experience play in guiding their statistical intuitions? We found that sequential experience—as opposed to text-based descriptions—of statistical information improved both children’s and adults’ reasoning in key inference problems. Moreover, experiencing statistical information boosted children’s and adults’ inferences to a similar extent. Indeed, although adults outperformed children in both description and experience, children’s experience-based inferences were superior to adults’ inferences in description. This result mirrors research on the “natural frequency facilitation effect” in Bayesian reasoning, showing that 12-year-olds’ inferences in a natural frequency format matched those of adults in a conditional probability format (Zhu & Gigerenzer, 2006).

To connect with existing work on the role of experience in improving adults’ statistical intuitions (e.g., Armstrong & Spaniol, 2017; Hogarth & Soyer, 2011; Wegier & Shaffer, 2017), we compared text-based descriptions of statistical information to the sequential experience thereof. Participants therefore had to be of at least elementary school age in order to read and understand the description-based problems. However, we would expect the beneficial effects of experience to be demonstrable for younger age groups as well. One important direction for future research is to compare younger children’s statistical intuitions in experience with age-appropriate descriptive formats. The remarkable inference abilities that have recently been demonstrated in preverbal infants and nonhuman animals (Denison & Xu, 2019; Rakoczy et al., 2014) support the idea that experiential formats are conducive to appropriate statistical inferences for even the youngest learners. We (Schulze & Hertwig, 2021) recently argued that the description–experience dimension is key to resolving the striking discrepancy between research suggesting that babies are born intuitive statisticians and research deeming adults’ statistical inferences to be in conflict with even “the simplest and the most basic qualitative law of probability” (Tversky & Kahneman, 1983, p. 293). In studies, infants must *experience* the statistical texture of the experimental microworld; in contrast, studies with older children and adults typically feature description-based and symbolic problem representations. This systematic and prevalent difference may contribute to the paradoxical impression of infant competence and adult failure in statistical inferences (Schulze & Hertwig, 2021). Had we contrasted adults’ statistical inferences in description with children’s inferences in experience in the present study (see dashed lines in Fig. 2), we would have concluded that children outperformed adults on two paradigmatic reasoning skills. Rather than indicating a developmental reversal, however, this result can be attributed to experimental protocol.

What might be the mechanisms underlying the beneficial effect of experience for children’s and adults’ statistical inferences? Several mechanisms have been suggested by which experience can facilitate appropriate statistical inferences, including by reducing computational difficulty, allowing for incremental learning, and enabling reliance on internal states as a source of information (e.g., Schulze & Hertwig, 2021). In our study, sampling experience may have improved children’s and adults’ inferences by easing computational demands. In conjunction problems, the sequential exposure to conjunctive events illustrated their numerosity, thus likely disambiguating the mathematical context and the applicability of the conjunction rule (see Hertwig & Gigerenzer, 1999). In Bayesian problems, the sequential experience of observations relieved participants of the need to process and operate on conditional probabilities or Arabic numerals and allowed them to make accurate inferences based on their ability to count and combine relevant observations (cf. Gigerenzer et al., 2021)—an ability typically emergent at preschool age (Gelman & Gallistel, 1978). Nonnumeric static icon array formats have been argued to render Bayesian inferences computationally simpler via a similar mechanism and have been shown to elicit Bayesian intuitions in second-graders and fourth-graders (Gigerenzer et al., 2021), as well as to improve adults’ Bayesian reasoning (e.g., Brase, 2009). Future research should compare the effects of static pictorial or summed frequency formats to the effects of sequential experience.

Although experience improved children’s and adults’ inferences to a similar extent and potentially via a similar process, the cognitive mechanisms underlying their statistical reasoning may nevertheless be different (see, e.g., Carey, 2009; Reyna & Brainerd, 1995). Moreover, experience-based and description-based notions of probability may follow distinct developmental trajectories. Thus, another important question for future research is how cognitive mechanisms engaged by description and experience develop (see also Schulze & Hertwig, 2021). Experiential formats can require costly information acquisition that taxes working memory; these demands may contribute to age-related differences in decision making (Rakow & Rahim, 2010). In the Iowa gambling task, for instance, reducing memory demands on children aged 7–11 years by giving them descriptive information about choice options before they directly experienced outcomes considerably improved their performance (van Duijvenvoorde et al., 2012). Here, we reduced memory load by implementing a card-based sampling procedure in which the entire sample remained available throughout (see Vallée-Tourangeau et al., 2015) and providing additional summary statistics to participants (see Hogarth & Soyer, 2011).

The ability to deal with described probability formats also appears to change significantly during development. For instance, 5–7 year old children do not systematically consider differences in expected value in risky choice when asked to choose between options with stated outcomes and probabilities, whereas children aged 8–11 years begin to approach the performance of adults (Levin et al., 2007). Such age-related differences in choice under known risk have been attributed to differences in working memory, suggesting that the ability to deal with described probabilities in making decisions is contingent on the maturation of cognitive control processes (Kray et al., 2021). More generally, understanding and executing described proportional calculations of probability appears to be a difficult skill for children to learn (Bryant & Nunes, 2012) and may require formal instruction. Fischbein and Gazit (1984) devised a teaching program for children aged 10–13 years on basic concepts of probability, including how to calculate the probabilities of simple and compound events. They found that children’s ability to understand these concepts and use them in a written test increased sharply with age.

In sum, experience is a key factor in determining the accuracy of both children’s and adults’ statistical intuitions. In highlighting the significant role of experience in shaping children’s inferences under uncertainty, this work has important implications for education policy. The description–experience distinction in risky choice has already fed into policy, for example, in the areas of risk communication, economic market design, and safe workplace practices (e.g., Erev & Roth, 2014; Hertwig & Wulff, 2021; Yechiam et al., 2006). It can also be harnessed to enrich formal education (Schulze et al., 2021) and, ultimately, may help determine how early experience-based strategies for dealing with uncertain information can support more formal views of probability taught in school.

## Appendix 1: Pilot study

To identify conjunction and Bayesian problems suited to both children and adults, we conducted a pilot study for which we recruited 10 children (age 8–11 years, mean age 9.20 years, four female) and 10 adults (age 21–37 years, mean age 28.10 years, four female) via the subject pool of the Max Planck Institute for Human Development. In this pilot study, we included inference problems that had previously been used with children in our target age range and that elicited large numbers of adequate inferences. For Bayesian reasoning, we adapted the natural frequency format of the five problems by Zhu and Gigerenzer (2006) that yielded at least 30% accuracy in a sample of adult participants (see Pighin et al., 2017). For reasoning about conjunctive probabilities, we adapted three problems involving social stereotypes (as in the paradigmatic Linda problem; for nonsocial conjunction problems used with children, see, e.g., Chiesi et al., 2008; Fisk & Slattery, 2005) with which children of our target age range would likely be familiar.^4^ Appendix Table 2 lists all inference problems used in the pilot study with their sources.

**Table 2:** Summary of inference problems used in the pilot study

Inference problem	Original study	Scenario name	Proportion of rule-consistent/correct responses		
			Children	Adults	Mean
Conjunction problem	Morsanyi and Handley (2008)	Sarah	0.90	1.00	0.95*
Conjunction problem	Morsanyi et al. (2017)	Tom	0.60	1.00	0.80*
Conjunction problem	Davidson (1995)	Mrs. Hill	0.30	1.00	0.65
Bayesian problem	Zhu and Gigerenzer (2006)	Red cards	0.50	0.90	0.70*
Bayesian problem	Zhu and Gigerenzer (2006)	Gloves	0.50	0.60	0.55*
Bayesian problem	Zhu and Gigerenzer (2006)	University students	0.30	0.70	0.50
Bayesian problem	Zhu and Gigerenzer (2006)	Headaches	0.30	0.70	0.50
Bayesian problem	Zhu and Gigerenzer (2006)	Red noses	0.10	0.80	0.45

*Note.* * Inference problems selected for the main study

The following adaptations were made to the original problems. The five Bayesian problems were modified to use first-person phrasing; to feature numerical information that could be sampled swiftly (i.e., total population sizes smaller than or equal to 40) but that preserved similar base rates, hit rates, and false-alarm rates (see Hafenbrädl & Hoffrage, 2015); and to include icon arrays that graphically displayed the numerical information. The three conjunction problems were modified to a frequency format (e.g., Hertwig & Gigerenzer, 1999) and included explicit frequency information about a subset of the total population to which the problem referred (e.g., participants were asked to imagine 100 children who fitted a particular stereotype but received frequency information about 20 randomly selected children; see also Morsanyi et al., 2017). We also included icon arrays that pictorially summarized the information. We relied on icon arrays rather than a sampling procedure in the pilot study to be able to test more scenarios within a given time frame without overtaxing children’s attention span. Under the assumption that description and experience represent concepts spread across a continuum (e.g., Rakow & Newell, 2010; Schulze & Hertwig, 2021), we understand natural frequencies and icon arrays as descriptive formats that reflect part of the underlying original experience (see, e.g., Gigerenzer & Hoffrage, 1995).

In sum, participants in the pilot study made a total of eight inferences (five Bayesian and three conjunction problems), earning €0.50 for each correct inference and an additional flat fee for their participation. Appendix Table 2 summarizes the proportion of correct Bayesian responses and responses consistent with the conjunction rule for each problem, separately for adults and children. For the Bayesian problems, we used a strict outcome criterion, counting only those responses as correct that numerically exactly matched the Bayesian solution (rounded to the nearest integer; see Zhu & Gigerenzer, 2006).

## Appendix 2: Reasoning problems used in the experiment

Appendix Table 3 summarizes all conjunction and Bayesian problems used in the experiment in both their description-based and experience-based formats.

**Table 3:** Child-friendly conjunction problems and Bayesian problems in both administration formats: Description-based (probability) and experience-based (sampling)

Description-based format	Experience-based format
Conjunction Problem 1	
Tim lives in a house with a large garden. His favorite school subject is playtime. He has many friends, and he loves sport. He collects soccer stickers. Mark the following statements with numbers 1 to 3 according to how likely they are to be true. Mark the statement which is the most likely to be true with 1, and the one which is the least likely to be true with 3. Tim plays soccer. [P] Tim plays an instrument. [LP] Tim plays soccer and an instrument. [C]	Tim lives in a house with a large garden. His favorite school subject is playtime. He has many friends, and he loves sport. He collects soccer stickers. Imagine 100 boys who are like Tim and who signed up for a summer camp. You can now meet some of these boys, who were picked at random for an assignment. Each card shows one randomly selected boy. The card also shows you what the boy likes to do. Some boys play soccer, others play an instrument, some play both, and some play neither. *----- sampling phase -----* You have now seen that out of every 20 boys who are like Tim, 12 play soccer, 3 play an instrument, and 2 play both soccer and an instrument. You can look at the cards for as long as you like. You can also rearrange them to understand the information as well as possible. When you are ready, imagine you meet a group of 100 boys who are like Tim. In other words, they live in houses with a large garden, their favorite school subject is playtime, they have many friends, they love sport, and they collect soccer stickers. What do you think: How many of the 100 boys play soccer? __ of 100 How many of the 100 boys play an instrument? __ of 100 How many of the 100 boys play soccer and an instrument? __ of 100
Conjunction Problem 2	
Sarah is 12. She is very talkative and sociable. She goes to drama classes, and she learns to play the guitar. She wants to be a pop singer or an actress. Mark the following statements with numbers 1 to 3 according to how likely they are to be true. Mark the statement which is the most likely to be true with 1, and the one which is the least likely to be true with 3. Sarah collects CDs. [P] Sarah likes to cook. [LP] Sarah likes to cook and collects CDs. [C]	Sarah is 12. She is very talkative and sociable. She goes to drama classes, and she learns to play the guitar. She wants to be a pop singer or an actress. Imagine 100 girls who are like Sarah and who signed up for a summer camp. You can now meet some of these girls, who were picked at random for an assignment. Each card shows one randomly selected girl. The card also shows you what the girl likes to do. Some girls collect CDs, others like to cook, some do both, and some do neither. *----- sampling phase -----* You have now seen that out of every 20 girls who are like Sarah, 8 collect CDs, 2 like to cook, and 1 likes to cook and collects CDs. You can look at the cards for as long as you like. You can also rearrange them to understand the information as well as possible. When you are ready, imagine you meet a group of 100 girls who are like Sarah. In other words, they are very talkative and sociable, they go to drama classes and learn to play the guitar, and they want to be a pop singer or an actress. What do you think: How many of the 100 girls collect CDs? __ of 100 How many of the 100 girls like to cook? __ of 100 How many of the 100 girls like to cook and collect CDs? __ of 100
Bayesian Problem 1	
During a cold winter, the probability that people in a town hurt their hands by the cold is 40%. If a person has hurt their hands, the probability that they wear gloves outside is 90%. If the person has healthy hands, the probability that they wear gloves is 40%. Imagine you meet a person from the town who wears gloves. What is the probability that the person has hurt their hands? [60%]	During a cold winter, some people in a town hurt their hands by the cold and others did not. Of the people who have hurt their hands, some wear gloves outside. Of the people with healthy hands, some also wear gloves. You can now meet some people from this town. Each card shows one of 25 people who were picked at random. The card also shows you whether this person has hurt their hands or has healthy hands, and whether the person wears gloves or not. *----- sampling phase -----* You have now seen that 10 out of every 25 people hurt their hands by the cold. Of the 10 people who hurt their hands, 9 wear gloves outside. Of the remaining 15 people with healthy hands, 6 also wear gloves. You can look at the cards for as long as you like. You can also rearrange them to understand the information as well as possible. When you are ready, imagine you meet a group of people from the town who wear gloves. How many of them hurt their hands? ___ out of ___. [9 out of 15]
Bayesian Problem 2	
A group of children is playing a card game. Those children who get a playing card with a picture of a cat on the front win a piece of candy. The probability that a playing card has a cat picture on the front is 25%. If a playing card has a cat picture on the front, the probability that it has a red back is 40%. If the playing card does not have a cat picture on the front, the probability that it has a red back is 53%. Imagine you draw a red playing card. What is the probability that it will have a cat picture on the front? [20%]	A group of children is playing a card game. Those children who get a playing card with a picture of a cat on the front win a piece of candy. Some playing cards have a cat picture on the front and others have no cat picture. Of the playing cards with a cat picture, some have a red back. Of the playing cards without a cat picture, some also have a red back. You can now look at some playing cards. Each card shows one of 20 playing cards that were picked at random. The card also shows you whether this playing card has a cat picture on the front or not, and whether the playing card has a red back or not. *----- sampling phase -----* You have now seen that 5 out of every 20 playing cards have a cat picture on the front. Of these 5 playing cards with a cat picture, 2 have a red back. Of the remaining 15 playing cards without a cat picture, 8 have a red back. You can look at the cards for as long as you like. You can also rearrange them to understand the information as well as possible. When you are ready, imagine you draw several red playing cards. How many of them have a cat picture on the front? ___ out of ___. [2 out of 10]

*Note*. In the conjunction problems, participants in both conditions judged three hypotheses: a probable constituent (P), a less probable constituent (LP), and the conjoined hypothesis (C). For the Bayesian problems, correct answers are shown in square brackets

## Footnotes

1 For children, assignment aimed to ensure a balanced age and gender distribution across conditions; for adults, assignment was contingent on the availability of an experimenter to conduct the sampling procedure.

2 Exclusion from the analyses of participants who failed to identify the stereotypical constituent as most likely/frequent on one or both of the conjunction problems (three adults, 13 children) did not change any of the reported conclusions.

3 Based on previous findings (see Zhu & Gigerenzer, 2006), we did not expect children aged 8–11 years to be able to estimate Bayesian posterior probabilities in the description-based task format. To our surprise, three children answered the first Bayesian problem correctly (see Table 1). Further inspection of their data indicated that two of these children had answered both Bayesian problems by inverting the false-alarm rates (responding 60% and 47% to the first and second problems, respectively; see Appendix Table 3). Three adults also showed this response pattern. Excluding these five participants from the analyses did not change any of the reported conclusions.
